# Silencing Inhibits Cre-Mediated Recombination of the Z/AP and Z/EG Reporters in Adult Cells

**DOI:** 10.1371/journal.pone.0005435

**Published:** 2009-05-05

**Authors:** Michael A. Long, Fabio M. V. Rossi

**Affiliations:** The Biomedical Research Centre, University of British Columbia, Vancouver, British Columbia, Canada; National Institute on Aging, United States of America

## Abstract

**Background:**

The Cre-*loxP* system has been used to enable tissue specific activation, inactivation and mutation of many genes in vivo and has thereby greatly facilitated the genetic dissection of several cellular and developmental processes. In such studies, Cre-reporter strains, which carry a Cre-activated marker gene, are frequently utilized to validate the expression profile of Cre transgenes, to act as a surrogate marker for excision of a second allele, and to irreversibly label cells for lineage tracing experiments.

**Principal Findings:**

We have studied three commonly used Cre-reporter strains, Z/AP, Z/EG and R26R-EYFP and have demonstrated that although each reporter can be reliably activated by Cre during early development, exposure to Cre in adult hematopoietic cells results in a much lower frequency of marker-positive cells in the Z/AP or Z/EG strains than in the R26R-EYFP strain. In marker negative cells derived from the Z/AP and Z/EG strains, the transgenic promoter is methylated and Cre-mediated recombination of the locus is inhibited.

**Conclusions:**

These results show that the efficiency of Cre-mediated recombination is not only dependent on the genomic context of a given *loxP*-flanked sequence, but also on stochastic epigenetic mechanisms underlying transgene variegation. Furthermore, our data highlights the potential shortcomings of utilizing the Z/AP and Z/EG reporters as surrogate markers of excision or in lineage tracing experiments.

## Introduction

**Figure 1:**
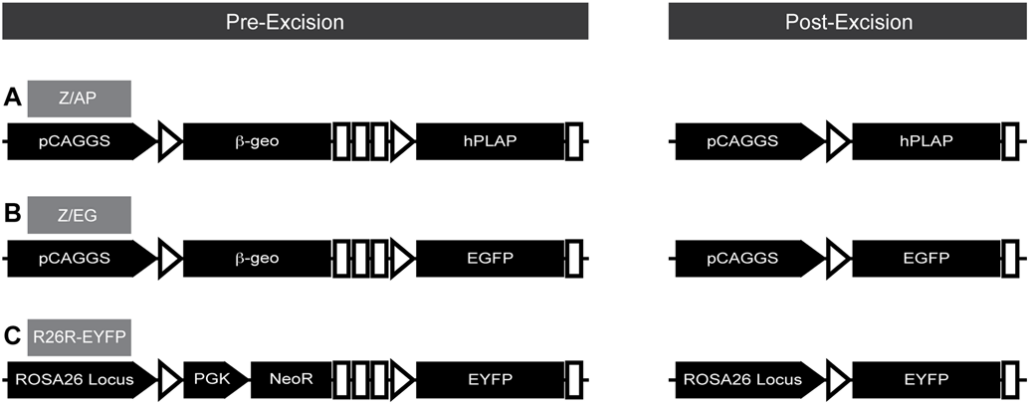
Genomic organization of Cre-reporter transgenes. In the Z/AP strain (A) and Z/EG strain (B), transcription of a β-galactosidase/neomycin phosphotransferase fusion gene (β-geo) is driven by a hybrid CMV/β-actin promoter (pCAGGS) and terminated by polyadenylation sites (white boxes). Cre recombines *loxP* sites (white triangles) to remove the β-geo gene, activating expression of human placental alkaline phosphatase (hPLAP) or enhanced green fluorescent protein (EGFP) in the Z/AP and Z/EG strains respectively. In the R26R-EYFP strain (C), Cre-mediated recombination of *loxP* sites removes a phosphoglycerate kinase (PGK) promoter and a neomycin phosphotransferase gene (NeoR), allowing the endogenous ROSA26 genomic locus to drive expression of the downstream enhanced yellow fluorescent protein (EYFP) gene.

The bacteriophage P1 enzyme, Cre, recognizes and recombines two copies of a specific 34 base-pair sequence known as a *loxP* sequence located at each end of the phages linear genome. This process converts the genome to a unit-copy circular plasmid ensuring proper replication and partitioning of the prophage [1], [2], [3]. This process does not require cofactors, therefore *loxP* sites inserted into the murine genome are also recognized and recombined by Cre, resulting in excision, inversion or translocation of chromosomal sequence depending on the orientation and location of the *loxP* sites [4]. To date, hundreds of transgenic mouse strains have been created which express Cre under the control of tissue specific or inducible promoters (http://nagy.mshri.on.ca/cre/) and in combination with transgenic mice containing *loxP*-flanked alleles, have revolutionized the study of the genetic factors involved in a multitude of biological processes. In order to facilitate such studies, several so-called Cre-reporter strains have also been created [4]. These mice generally carry the gene for an easily detectable marker, the expression of which is only activated following exposure to Cre. For example, in the Z/AP reporter strain (Figure 1A), transcription of a β-galactosidase/neomycin phosphotransferase fusion gene (β-geo) is driven by a hybrid CMV enhancer/chicken β-actin promoter (pCAGGS) and terminated by a trimer of SV40 polyadenylation sites [5]. The β-geo gene and polyadenylation sites are flanked by *loxP* sites oriented in the same direction. Therefore, upon exposure to Cre, the β-geo gene is excised and transcription of the human placental alkaline phosphatase (hPLAP) gene is activated by proximity to the pCAGGS promoter. The Z/EG strain (Figure 1B) was derived from the Z/AP reporter and contains the cDNA for enhanced green fluorescent protein (EGFP) in place of hPLAP [6]. Thus, all cells of Z/AP and Z/EG mice are designed to exhibit a binary readout of Cre activity, expressing β-galactosidase by default and activating expression of hPLAP or EGFP respectively upon exposure to Cre. The R26R-EYFP strain (Figure 1C) was generated by targeted insertion of a Cre reporter cassette into the ROSA26 genomic locus, which has been shown to be ubiquitously expressed throughout development and in adult tissues [7], [8]. The reporter cassette contains a PGK promoter driving expression of a neomycin phosphotransferase gene, both of which are removed by Cre mediated recombination of flanking *loxP* sites. Following excision, the endogenous ROSA26 promoter drives expression of the downstream enhanced yellow fluorescent protein (EYFP) gene.

Cre-reporter strains have been utilized to validate the expression profile of Cre transgenes [9], to act as a surrogate marker for excision of a second allele [10], to irreversibly label cells for lineage tracing experiments [11] and to differentiate between fusion and transdifferentiation in studies of stem cell plasticity [12]. Although it is known that the chromosomal integration site of *loxP* sequences can affect the efficiency with which they are recombined [13], to date there has not been a systematic comparison of the labeling efficiencies of some of the most widely used Cre-reporter strains. We have undertaken such a comparison and have demonstrated that the efficiency of reporter activation in adult cells derived from the Z/AP and Z/EG strain is much lower than in adult cells derived from the R26R-EYFP strain. Furthermore, our evidence suggests that the inefficient labeling efficiency observed in the Z/AP and Z/EG strains is due to methylation of the pCAGGS promoter which prevents both reporter expression and Cre-mediated recombination of the transgenic locus.

## Results

**Figure 2:**
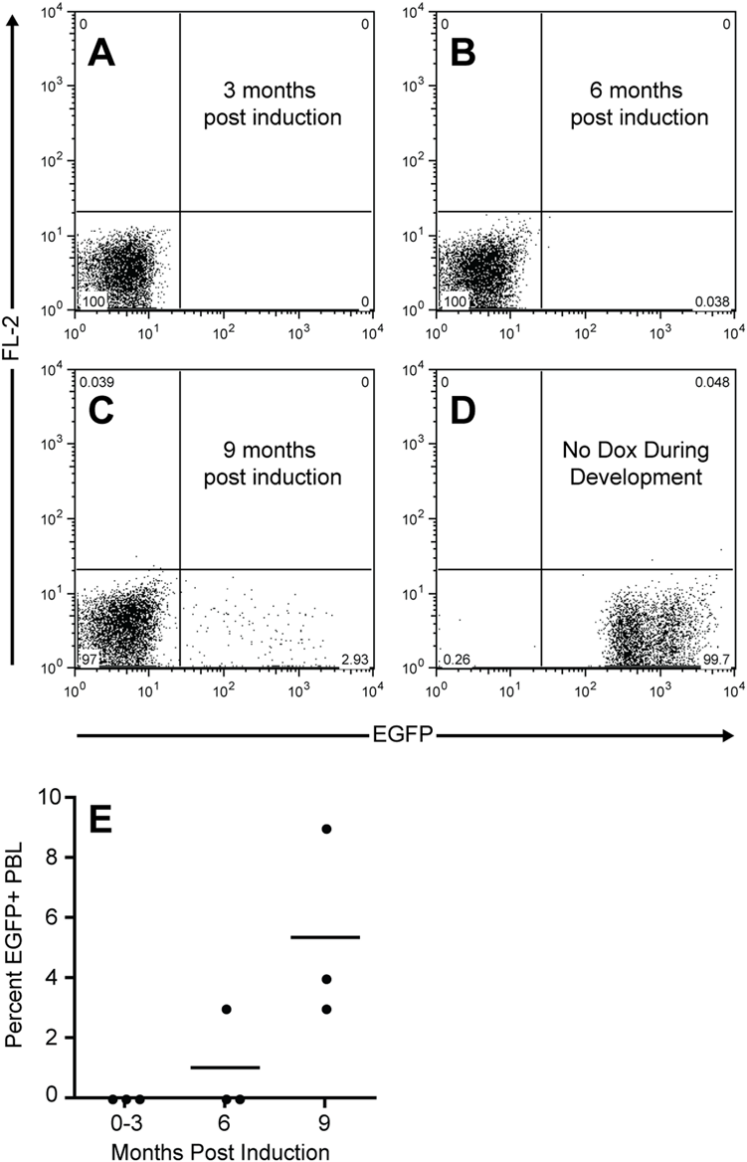
Activation of the EGFP reporter is inefficient in the blood of adult TIE2-tTA/Tet-O-Cre-/ZEG mice. EGFP expression in the blood of a representative triple transgenic mouse, 3, 6 and 9 months following removal of doxycycline from the diet is shown in (A–C) respectively. EGFP expression in the blood of a representative triple transgenic mouse, bred in the absence of doxycycline is shown in (D). In all triple transgenic mice analyzed, activation of the EGFP reporter was not observed in peripheral blood leukocytes (PBL) earlier than 6 months following removal of doxycycline and remained low at nine months post induction (E).

The Z/EG reporter strain has become a valuable tool for studying embryonic development [14], [15]. However, we were interested in utilizing this strain to study both embryonic and adult hematopoiesis and therefore created the triple transgenic TIE2-tTA/Tet-O-Cre/Z/EG strain. In these mice, expression of the tetracycline-transactivator is driven by the TIE2 promoter, which has been shown to be active in hematopoietic stem cells [16], [17]. Therefore following removal of doxycycline from the diet of mice, the tetracycline-transactivator is able to bind to the tet-operator and drive expression of Cre in hematopoietic stem cells, theoretically resulting in expression of the EGFP reporter in all hematopoietic lineages. In order to determine the kinetics of reporter activation in this system, we analyzed the blood of triple transgenic mice for expression of EGFP at several intervals following removal of doxycycline from the diet (Figure 2A–2C). Although EGFP positive cells were eventually detected in the blood of all triple transgenic mice, in the best case less than 10 percent of peripheral blood leukocytes were labeled following nine months of induction (Figure 2E). Conversely, the blood of triple transgenic mice that developed in the absence of doxycycline was labeled quite efficiently, validating that the system does function properly during development (Figure 2D). While this suggests that the Z/EG reporter may not function as well in adult hematopoietic cells as it is during development, TIE2 expression has been proposed to maintain hematopoietic stem cells in a quiescent state, thus it remains possible that in triple transgenic mice, TIE2 positive hematopoietic stem cells are labeled quite efficiently and simply do not contribute significantly to the peripheral blood [18]. Furthermore, although unlikely, it is possible that following removal of doxycycline from the diet of triple transgenic mice, the levels of the drug remaining in vivo are sufficient to suppress expression of Cre.

**Figure 3:**
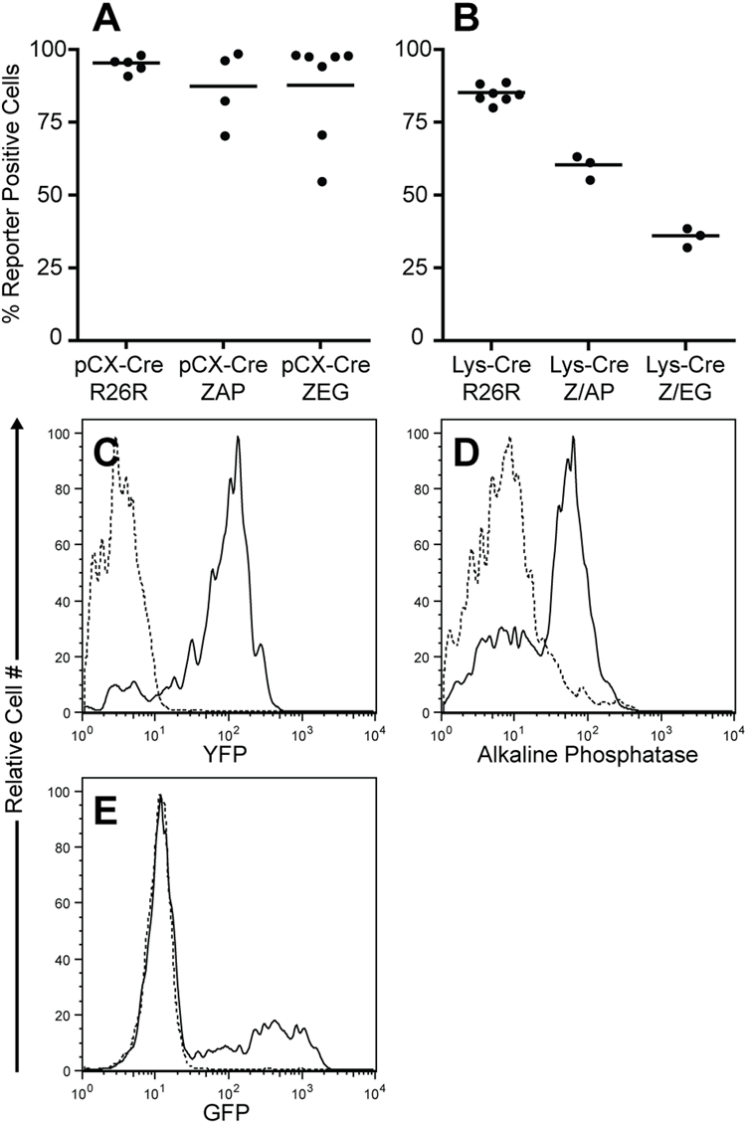
Activation of the Cre-reporter gene is less efficient in Z/AP and Z/EG mice than in R26R-EYFP mice. All reporter strains were crossed to the general deleter strain, pCX-NLS-Cre, and activation of each reporter gene was assessed by flow cytometry of peripheral blood leukocytes at 12 weeks of age (A). All reporter strains were also crossed to the myeloid-specific LysM-Cre strain, and activation of each reporter gene was assessed by flow cytometry of peripheral blood granulocytes at 12 weeks of age (B). Representative histograms demonstrating reporter expression in peripheral blood granulocytes are shown in (C–E) for LysM-Cre/R26R-EYFP, LysM-Cre/ZAP and LysM-Cre/Z/EG mice respectively (solid lines). Reporter expression in BL/6 mice is shown in dotted lines.

In an effort to eliminate pharmacological complications and to test the hypothesis that the Z/EG reporter does not function as efficiently in adult hematopoietic cells as it does during development, we crossed Z/EG mice to the LysM-Cre strain, which expresses Cre in all myelomonocytic cells and to the general deleter strain pCX-NLS-Cre [19], [20]. As a basis for comparison, LysM-Cre and pCX-NLS-Cre mice were also bred to the Z/AP reporter strain as well as to the R26R-EYFP reporter strain and peripheral blood leukocytes were analyzed for expression of the appropriate post-excision reporter. As expected, the R26R-EYFP reporter was activated in all peripheral blood leukocytes of pCX-NLS-Cre/R26R-EYFP mice (Figure 3A) and in 85 percent of granulocytes of LysM-Cre/R26R-EYFP mice (Figure 3B). A similar labeling efficiency of LysM-Cre/R26R-EYFP granulocytes has previously been reported and most likely approaches the maximum labeling efficiency of these short-lived cells utilizing a Cre transgene expressed from a promoter which is activated during their life-cycle [21]. In contrast to these results, the average labeling efficiency of granulocytes in the LysM-Cre/Z/AP and LysM-Cre/Z/EG strains was only 57 percent and 36 percent respectively (Figure 3B) and remained consistent at these levels over time (data not shown). A similar labeling efficiency has been reported for the Z/EG strain following a cross to a separate myeloid specific-Cre strain [9]. These data, taken together with the observation that it is possible to activate the hPLAP and EGFP reporter in virtually all blood cells of most pCX-NLS-Cre/Z/AP and pCX-NLS-Cre/Z/EG mice (Figure 3A), further supports the hypothesis that the Z/AP and Z/EG reporters do not function as efficiently in adult hematopoietic cells as they do during development. The presence of a significant percentage of hPLAP negative and EGFP negative cells in a subset of pCX-NLS-Cre/Z/AP and pCX-NLS-Cre/Z/EG mice (Figure 3A) has also been reported by others and demonstrates that even under conditions of embryonic exposure to high levels of Cre recombinase, the Z/AP and Z/EG reporters are not completely reliable [6], [12], [22].

**Figure 4:**
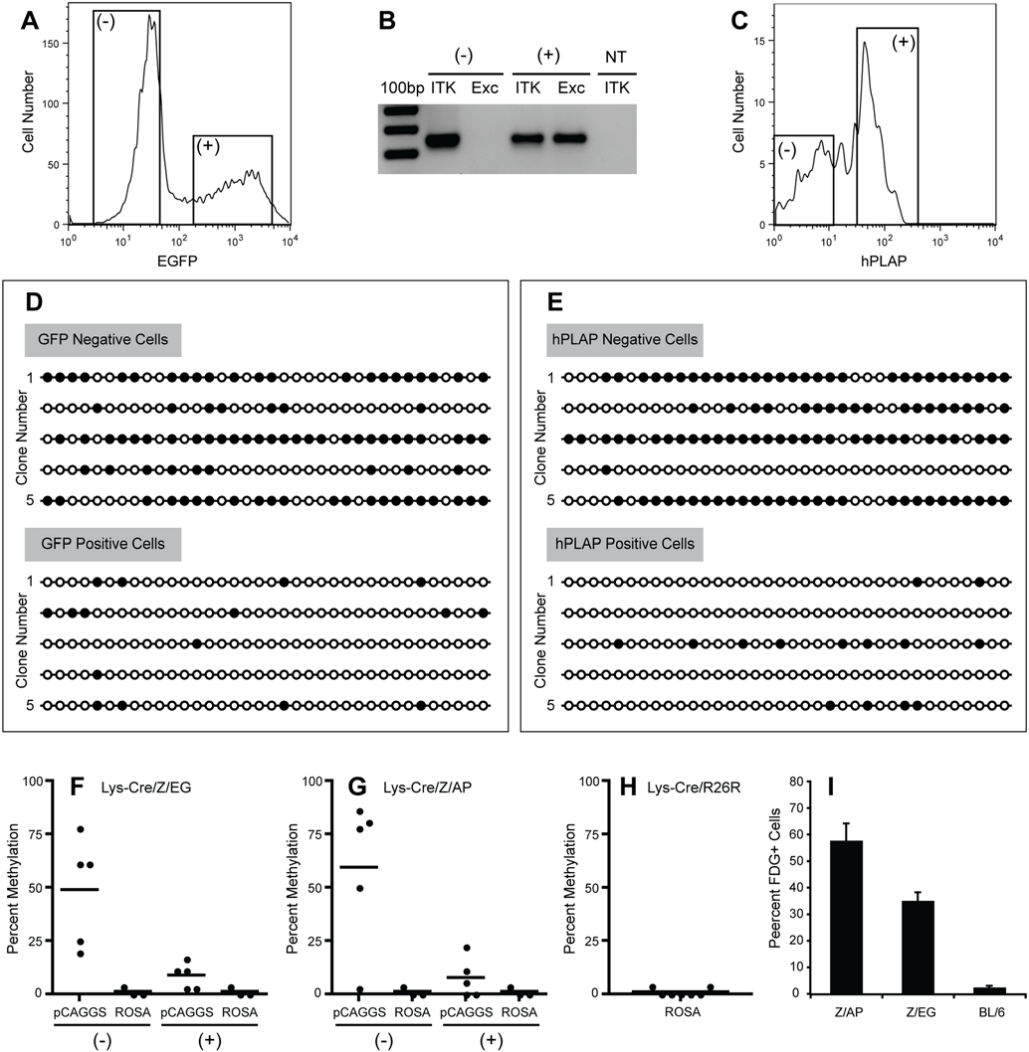
Mechanisms contributing to the inefficient labeling efficiency of Z/AP and Z/EG cells. Gr-1 positive cells from a LysM-Cre/Z/EG mouse were sorted into reporter negative (−) and reporter positive (+) populations (A). Genomic DNA from these populations was then analyzed by PCR utilizing primers binding within the pCAGGS promoter and EGFP cDNA. This combination of primers (Exc) generates a 240bp fragment in the presence of the recombined locus whereas the distance across the intact locus is too large to facilitate exponential amplification under the conditions used (B). Primers recognizing the IL-2 inducible T-cell kinase (ITK) gene were also utilized as a positive control in reporter negative (−) and reporter positive (+) reactions as well as in a no-template (NT) control. Gr-1 positive cells from a LysM-Cre/Z/AP mouse were also sorted into reporter negative (−) and reporter positive (+) populations (C). Genomic DNA from reporter negative and reporter positive populations was subjected to bisulfite sequencing in order to determine the methylation status of a 200 bp region of the pCAGGS promoter. This region contains 36 CpG dinucleotides, significantly more of which were methylated (filled circles) in clones derived from reporter negative cells than in those derived from reporter positive cells (D,E). The degree of methylation at the pCAGGS and ROSA promoters in both reporter negative (−) and reporter positive (+) populations is shown in (F) and (G) for cells derived from LysM-Cre/ZEG and LysM-Cre/Z/AP mice respectively. The degree of methylation at the ROSA promoter in cells derived from the LysM-Cre/R26R-EYFP strain is shown in (H). Silencing of the Z/AP and Z/EG transgenic loci was demonstrated via quantification of the percentage of peripheral blood leukocytes expressing β-galactosidase by FDG staining (I).

The differential labeling efficiencies observed in LysM-Cre/R26R-EYFP, LysM-Cre/Z/AP and LysM-Cre/Z/EG mice are unlikely to be due to variable expression of functional Cre recombinase as all mice contain the same LysM-Cre transgene and were maintained on the same genetic background. Therefore, we reasoned that the inefficient labeling of Z/EG and Z/AP granulocytes may be due to impaired recombination and/or inefficient expression of the transgenic loci. In order to differentiate between these two scenarios, we designed a PCR-based strategy to examine the efficiency of Cre-mediated excision of the β-geo gene. Granulocytes from LysM-Cre/Z/EG mice were first sorted into reporter-negative and reporter-positive populations (Figure 4A). Genomic DNA from these groups was then subjected to PCR reactions containing primers designed to generate an amplicon only in the presence of a recombined transgenic locus. As seen in Figure 4B, reporter positive cells contain a recombined locus as expected. In the reporter-negative population however, the recombined locus was not detected, suggesting that Cre may be unable to efficiently access the *loxP* sites in the genomic DNA of cells derived from the LysM-Cre/Z/EG strain.

As DNA methylation is known to be one of the primary mechanisms by which eukaryotic cells silence foreign DNA, we hypothesized that the Z/EG transgene is methylated and incorporated into heterochromatin in adult hematopoietic cells, thereby reducing the accessibility of the *loxP* sites for Cre mediated recombination [23], [24]. We therefore subjected a 200 base-pair segment of the pCAGGS promoter containing 36 CpG dinucleotides to bisulfite sequencing in order to examine its methylation status in both reporter-negative and reporter-positive granulocytes from the LysM-Cre/Z/EG strain. As seen in Figure 4D and 4F, this segment of the pCAGGS promoter is indeed methylated to a greater extent in reporter negative cells than it is in reporter positive cells. In order to demonstrate that hypermethylation is not a global phenomenon in reporter negative cells, we also determined the methylation status of a 253 base-pair segment of the endogenous ROSA26 promoter containing 28 CpG dinucleotides in both reporter-negative and reporter positive cells. As seen in Figure 4F, very little methylation was observed at this locus in all clones examined, regardless of reporter expression. A similar correlation between hypermethylation of the pCAGGS promoter and inefficient expression of the Cre-reporter gene was also observed in granulocytes obtained from LysM-Cre/Z/AP mice (Figure 4C, 4E, and 4G).

A corollary to our hypothesis that DNA methylation inhibits Cre-mediated activation of the reporter gene in the Z/AP and Z/EG strains is that the ROSA26 promoter should be unmethylated in granulocytes derived form the LysM-Cre/R26R-EYFP strain. As seen in Figure 4H, this is indeed the case.

As a final confirmation of the fact that the Z/AP and Z/EG loci are silenced in adult hematopoietic cells, we also quantified expression of the pre-excision reporter, β-galactosidase, via fluorescein-di-beta-D-galactopyranoside (FDG) staining of peripheral blood leukocytes taken from these mice. As seen in Figure 4I, the pre-excision reporter is also inefficiently expressed in adult hematopoietic cells of Z/AP and Z/EG mice presumably due to epigenetic silencing of the transgenic locus.

## Discussion

We have demonstrated a diminished sensitivity to Cre-mediated recombination in adult hematopoietic cells derived from Z/AP and Z/EG mice. These reporter cassettes were randomly inserted into the mouse genome and as such are more likely to be subjected to position effect variegation than the R26R-EYFP reporter, which was inserted into the ubiquitously expressed ROSA26 genomic locus. In accordance with this hypothesis we have also demonstrated that expression of the pre-excision reporter is variegated in adult hematopoietic cells derived from Z/AP and Z/EG mice. The difference in the degree of silencing observed between Z/AP and Z/EG cells is unlikely to be due to differences in the location or extent to which the pCAGGS promoter is methylated as these parameters appear to be quite similar between cells derived from the two strains (Figure 4D–4G). Thus, differences in the chromatin conformation of the loci into which the transgenes have been inserted likely influence the frequency and not the extent to which the pCAGGS promoter is subjected to methylation, underlying the observed differences in silencing.

Interestingly, the percentage of cells expressing the pre-excision reporter (Figure 4I) in both Z/AP and Z/EG mice is remarkably similar to the percentage of cells expressing the post-excision reporter (Figure 3B) in LysM-Cre/Z/AP and LysM-Cre/Z/EG mice respectively. This observation suggests that the population of cells that express the pre-excision reporter may be the only cells capable of undergoing Cre-mediated activation of the post-excision reporter in adult granulocytes.

In order to explain these findings, we propose a model wherein the Z/AP and Z/EG loci are demethylated after fertilization as a result of the genome wide demethylation that is known to occur in pre-implantation embryos [25]. Therefore, if these transgenes are exposed to Cre recombinase shortly after fertilization, such as by crossing to the pCX-NLS-Cre strain, which ubiquitously expresses Cre, the locus is accessible, the β-geo gene is efficiently excised and the downstream reporter is expressed. As embryonic development progresses however, the Z/AP and Z/EG transgenes become methylated, resulting in the eventual incorporation of the transgenic loci into heterochromatin, which inhibits access of transcription factors and Cre recombinase. Therefore, if these transgenes are exposed to Cre recombinase in adult cells, such as by crossing to the LysM-Cre strain, the β-geo gene is inefficiently excised and the locus is inefficiently expressed. The presence of the β-galactosidase (LacZ) sequence within the β-geo gene may be a significant contributor to this effect as the CpG-rich LacZ cDNA is known to induce silencing of some genes to which it is fused [26]. In accordance with this notion, we have demonstrated that excision of the β-geo gene shortly after fertilization, significantly reduces silencing of the reporter loci in adult cells (Figure 3A).

Although we have restricted our analysis to the labeling efficiency of hematopoietic cells, other groups have also reported low labeling efficiency utilizing the Z/EG strain in the adult kidney, liver, testis, adrenal glands, fat tissue, lung, pituitary gland, spleen and retina [27], [28]. Furthermore, Rotolo *et al*. have recently developed a method for the analysis of neuronal morphology, which is based in part on the inefficiency with which the Z/AP locus is recombined in the adult brain [29]. Our findings highlight the potential shortcomings of utilizing these particular Cre-reporters as surrogate markers of excision or in lineage tracing experiments. Therefore, the R26R-EYFP reporter may be the strain of choice for researchers interested in tracing the expression of Cre beyond early development.

## Materials and Methods

### Ethics Statement

All experiments were performed in accordance with the rules of the Animal Care Committee at the University of British Columbia.

### Transgenic Mice

The Z/AP, Z/EG, pCX-NLS-Cre, TIE2-tTA and Tet-O-Cre strains were generously provided by Dr. Corrinne Lobe and the LysM-Cre and R26R-EYFP mice were generously provided by Dr. Thomas Graf. Mice were housed in a specific pathogen free facility and each strain was maintained by backcrossing to the C57BL/6 strain.

### TIE2-tTA/Tet-O-Cre/Z/EG Mice

Double transgenic TIE2-tTA/Tet-O-Cre mice were crossed with Z/EG mice and breeders were fed Dox-Diet (Bio-Serv) containing 200 mg/kg doxycycline. After weaning, triple transgenic pups were maintained on Dox-Diet until 8 weeks of age at which time doxycycline was removed from the diet. Peripheral blood then was taken daily for a week, weekly for a month and at 2, 3, 6 and 9 months post induction. Peripheral blood leukocytes were analyzed by flow cytometry for the expression of the Cre-reporter transgene, EGFP.

### Flow Cytometry

Peripheral blood samples were taken from the tail vein of each mouse and erythrocytes were lysed in a hypotonic solution. For experiments requiring identification of granulocytes or hPLAP, cells were stained with a PE-conjugated anti-Gr-1 antibody (eBioscience) or an anti-human hPLAP antibody (Serotec) respectively. For FDG staining, blood cells were incubated in a hypotonic solution containing 1 mM fluorescein-di-beta-D-galactopyranoside for 1 minute at 37°C. The mixture was then diluted 10-fold in PBS and incubated for 1 hour on ice. All data was collected with a Becton-Dickinson FACSCalibur and analyzed with FlowJo software.

### Excision Analysis

Peripheral blood samples were prepared and stained with a PE-conjugated anti-Gr-1 antibody as described above. Reporter negative and reporter positive granulocytes were sorted utilizing a Becton-Dickinson FACSVantage and genomic DNA was prepared from roughly 3×10^4^ sorted cells from each population (DNeasy Blood and Tissue Kit, QIAGEN). PCR primers designed to amplify a segment of the ITK gene were as follows: ITK-F: 5′-GCCGTAAATGAACAGGTGGTG-3′ and ITK-R: 5′-TGCTCCAGACTGTGAGAGTCG-3′. Primers designed to identify a recombined Z/EG locus were pCAGGS-F: 5′-GGGCAACGTGCTGGTTGT-3′ and EGFP-R: 5′-CCAGCTCGACCAGGATGG-3′.

### Bisulfite Sequencing

Genomic DNA from reporter negative and reporter positive populations was converted utilizing the EpiTect Bisulfite Kit (QIAGEN). For analysis of the pCAGGS promoter, DNA was subjected to a semi-nested PCR reaction utilizing primers BABF6: 5′-GGAGAGGTGYGGYGGTAGTTAATTAGAG-3′ and BABR5d: 5′-AAACCCCTCAAAACTTTCACRCAACCACAA-3′ for the first round followed by BABF6 and BABR4c: 5′-TCATTAAACCAAACRCTAATTACAACCC-3′ for the second round. Analysis of the ROSA26 promoter utilized the primers ROSAF2: 5′-GGAAAYGTTATTGATYGTAYGGGGATT-3′ and ROSAR3: 5′-ACTATCTCACAAAACRACTCCACCAC-3′. PCR products were cloned into the pCR2.1 vector (Invitrogen) and sequenced from the T7 priming site utilizing Applied Biosystems BigDye v3.1 Terminator Chemistry at the NAPS Unit, UBC.
